# Polycapillary Optics for Materials Science Studies: Instrumental Effects and Their Correction

**DOI:** 10.6028/jres.109.003

**Published:** 2004-02-01

**Authors:** M. Leoni, U. Welzel, P. Scardi

**Affiliations:** Università di Trento, Dipartimento di Ingegneria dei Materiali e Tecnologie Industriali, Via Mesiano 77, 38050 Trento ( Italy); Max Planck Institute for Metals Research, Heisenbergstr. 3, 70569 Stuttgart ( Germany); Università di Trento, Dipartimento di Ingegneria dei Materiali e Tecnologie Industriali, Via Mesiano 77, 38050 Trento ( Italy)

**Keywords:** instrumental effects, parallel beam geometry, polycapillary optics, residual stress analysis, texture analysis, x-ray diffraction, x-ray optics

## Abstract

The instrumental effects related to the use of a polycapillary x-ray lens as primary beam collimator are here studied and the features observed in the measurements modelled via Monte-Carlo ray-tracing. Comparison with existing procedures is presented and experimental evidence of the accuracy improvements due to the use of a correction algorithm is shown.

## 1. Introduction

Fast data collection is a primary need in experimental crystallography. A high throughput can be obtained using third generation synchrotrons and high-flux neutron sources, but their availability, accessibility and cost is far beyond the required figures.

The need is therefore for more brilliant laboratory sources: besides the use of more powerful x-ray generators, the primary beam flux can be increased by means of last-generation optical devices such as multilayer mirrors (a.k.a. Göbel mirrors) and polycapillary collimators (a.k.a. Kumakhov optics). Multilayer mirrors are composed of alternating layers of heavy and light elements: an incoming beam is enhanced by the constructive interference among the various wave fronts produced by its reflection on the layered structure. In a polycapillary device, the x-rays are funneled through narrow glass capillaries by total external reflection at the capillary walls. These two broad classes of devices are, to a certain extent, complementary; multilayer mirrors are more suited for applications where a line focus is required (reflectometry, grazing incidence diffraction, etc.), whereas a polycapillary lens suits a point focus configuration (e.g., stress and texture analysis). Both types of optical devices can be built as to impose a focused or a parallel character to the beam and to provide specific filtering properties.

Despite their widespread availability, both optical component are not exhaustively described in the x-ray diffraction literature: in the present work, features and instrumental effects of polycapillary collimators will be analyzed in detail by using a Monte-Carlo ray-tracing approach.

### 1.1 Polycapillary Lenses

The idea of using straight capillaries to steer an x-ray beam to the specimen, thus increasing the effective flux, dates back to the 1950s (e.g., Refs. [1–2]). Capillaries were tested not only to guide but also to squeeze the x-rays to a very small spot. Eventually, the experimentation conducted on laboratory instruments moved to synchrotron sources, where the use of these devices would provide a bright collimated beam [3–10]. Applications steadily increased and detailed studies on the optical response were conducted; for instance the original conical tapering was soon abandoned in favor of ellipsoidal or parabolic ones [6] that guarantee better optical properties of the produced beam. However, the development of devices resembling the present-day collimators, started in the 1980s in the former Soviet Union and actual prototypes were presented in the 1990s [11–13]. It is therefore in the last decade that literature and applications of these new devices had a considerable increase.

Polycapillary optics act as x-ray guides to funnel the rays from the point focus of a tube to the surface of the specimen; funneling is achieved by multiple total reflection of the rays on the inner walls of hollow glass fibers. Tapered and curved capillaries of circular, square or hexagonal cross section can be tailored to the users’ needs to focus or straighten the x-ray beam. Moreover, since the funneling principle is also applicable to neutron beams, the development of neutron optics has paralleled that of x-ray optics [12–15]. Polycapillary devices act also as angular and energy filters since the critical angle above which total reflection does not occur is energy-dependent [16]; in particular, the divergence of the beam is determined both by the critical angle (i.e., energy of radiation and constitutive material) and by the diameter and length of the capillaries.

Older devices and neutron beam collimators use single capillary or polycapillary fibers guided through metal meshes, whereas for laboratory use the capillary fibers are closely packed along their entire length (monolithic Kumakhov optic) and tapered to the desired shape.

### 1.2 Stress/Texture Measurements for Materials Analysis

The knowledge of the residual stress state in technological components is essential to assess their reliability and durability, and to guarantee the quality of manufactured products. Developed since the twenties, the techniques for the measurement of orientation and residual stress in bulk materials and thin films using x-ray diffraction can nowadays profit from the availability of dedicated diffractometers. However, major issues still remain precision and accuracy, closely related to the signal-to-noise ratio.

The most used technique for stress analysis is the so-called “sin^2^*ψ* method” [17–18], based on the collection of diffraction data at various tilts of the specimen about the axis perpendicular to the scattering direction and lying in the equatorial plane (*ψ* angle). Instrumental errors due to specimen tilting should be thus carefully considered. Quite often, most of the information regarding the stress state in the measured specimen (in particular, stress gradients) is contained in the high-tilt part of the sin^2^*ψ* region where instrumental aberrations play the major role. It is not infrequent the case where, owing to texture, data can be collected across limited angular ranges at high *ψ* angle, raising serious doubts on the reliability of the analysis if due correction is not made.

Correction procedures are not always available for the chosen experimental setting; most commercially available software packages can deal only with a circular beam and with specimens of round shape or such that the whole primary beam is intercepted for all values of *ψ* and 2*θ*, conditions not always met in normal laboratory practice (e.g., they can be violated in presence of a specimen displacement or at high tilting). When a square beam is available (crossed slit collimator) or, in general when high-resolution is requested, the reliability of these corrections is doubtful. Simple procedures for correcting instrumental effects when a polycapillary lens is used for pole figure measurements have been recently presented by Welzel and Leoni [19].

## 2. Experimental Set-Up

**Fig. 1:**
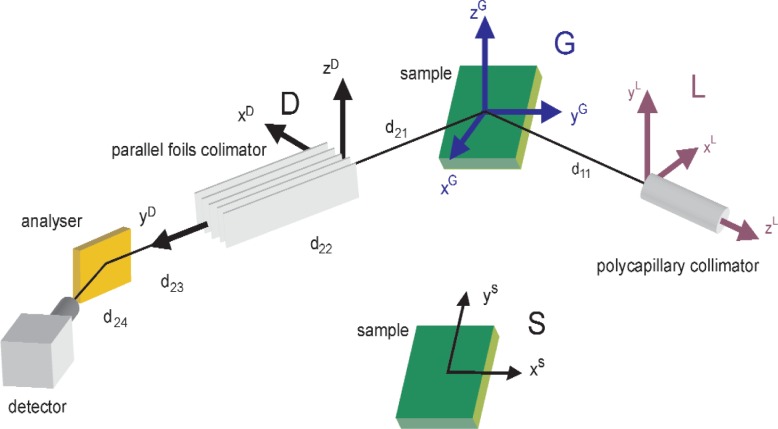
Details of the beam path with indication of the optical components and reference systems used throughout the paper. The references G and L should be concentric (same origin; the latter has been shifted for clarity).

Measurements have been conducted on two Philips X’Pert MRD 4-circle diffractometers^1^ (in the following identified as MRD1 and MRD2, respectively). Both machines are operated by long fine focus copper tubes (maximum power 2.2 kW) in point focus mode and have the same optical setup with a polycapillary collimator followed by a set of adjustable crossed-slits in the primary path and a parallel foils collimator plus a graphite flat-crystal analyzer on the secondary arm. The nominal (outer) diameter of the polycapillary lens was 6 mm and 9 mm for the two instruments, respectively. More details on the actual beam path are given in Fig. 1 and in the raytracing section.

To characterize both instrumental aberrations and features of the correction algorithm, a large set of specimens was used:

- Fine ground tungsten (Merck) and germanium (Johnson-Mattey) powders. The specimens are analogous to those used in [19] and were obtained by filling a shallow square cavity cut on a flat aluminum disk. Particular care was taken to assure the flatness of the surface of the powder, checked by means of an optical microscope. The dimension of the cavity was 14 mm × 14 mm for both specimens. The samples can be considered as infinitely thick (real thickness 2 mm);
- Fine ground silicon powder (Ventron). The specimen was the same as in the cited paper by Welzel and Leoni [19], and was obtained by sedimentation of the powder, previously dispersed in ethanol, onto a silicon wafer on a 14 mm × 14 mm area as to obtain an average mass coverage of 9.4 µg/mm^2^;
- Copper thin film. A thin copper layer (500 nm) was deposited onto an oxidized silicon substrate in ultra high vacuum by magnetron sputtering (further details can be found in [20]). This specimen, possessing a strong but complex texture, was used to check the performance of the raytracing algorithm for the correction of the instrumental effects;
- Cold rolled Ni(V) sheet (a typical metal substrate for high-*T*_c_ superconducting thin films); the sheet was rolled to induce a high degree of in-plane texture;
- Zinc oxide powder (ZnO, Carlo Erba Analyticals) for the evaluation of the instrument broadening function. A line profile standard (such as LaB_6_, SRM 660a) should be used for the characterization of the instrumental function for the various diffractometers. However, due to the low resolution expected for the instrument (i.e., wide peaks), the residual broadening of a fine ground zinc oxide powder is negligible with respect to the instrumental width thus virtually any kind of fine ground powder could be used. Moreover, zinc oxide forms very flat surfaces and the powder aggregate is compact enough to permit measurements at positive and negative tilting. The powder was loaded in a sample holder equal to that used for tungsten and silicon;
- Lanthanum hexaboride standard powder, LaB_6_ SRM 660a. This is the line profile/line position standard recently produced by the National Institute of Standards and Technology (NIST) [21]; it possesses negligible size and strain broadening and it will be used for comparison with the ZnO powder.

Some data regarding these specimens will be presented here. A custom non-linear least squares fitting program based on Pearson VII (PVII) functions was used to extract peak position and shape information from the raw data [19]. The emission profile was considered as a doublet of PVII functions with bound shape parameters and positions (for the emission spectra of copper, see for instance [22]). For each diffraction line or group of overlapping diffraction lines, a linear background was assumed.

## 3. Modeling of the Lens/Crossed-Slit Assembly

### 3.1 Beam Divergence

When a polycapillary collimator is used, the divergence of the primary beam (both axial and equatorial) is mainly determined by the diameter of the capillaries, their tapering and the type of glass employed in the fabrication. The knowledge of the angular dispersion of the primary beam is of great importance for a correct modeling of the diffraction system. The equatorial divergence can be measured by scanning the primary beam about the 2*θ* = 0° position and using a narrow crossed slit placed in front of the detector. In the same way, the axial divergence should be measured by scanning the primary beam perpendicularly to the diffraction plane, a motion not attainable even on a 4-circle diffractometer, since source and detector cannot move out of the equatorial plane.

**Fig. 2:**
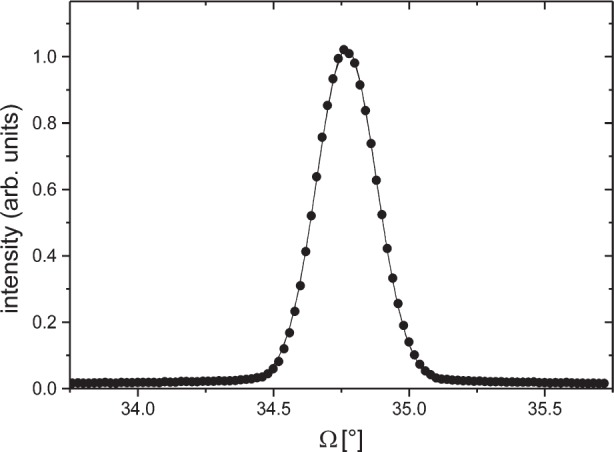
(004) Si rocking curve (tilt about the L*_z_* axis) of a <00*l*> cut silicon wafer collected on MRD I: experimental values (dots) and gaussian fit (line).

An alternative and sufficiently accurate method for measuring the equatorial divergence consists in collecting a rocking curve (*ω* scan) about one of the reflections of a single crystal. Figure 2 shows the (004)-Si rocking curve of a silicon wafer (<00*l*> cut) obtained at 45 kV and 40 mA with completely open slits and without any secondary optics but an aluminum foil (attenuator) placed in front of the detector. When the (intrinsic) Darwin width of the specimen is negligible with respect to the beam divergence, the Full Width at Half Maximum (FWHM) of the rocking curve (indicated in the following as *α*) is a good estimate for the equatorial divergence. In this case *α* ≈ 0.3°, thus the approximation is fully justified (the Darwin width for a silicon wafer is two orders of magnitude smaller).

Due to the difficulties in measuring the axial divergence accurately and since there is no reason to suppose axial and equatorial divergence to differ (the capillaries are circular and the whole assembly possesses a cylindrical symmetry), the two divergences will be considered equal at the exit of the collimator.

In the secondary path the divergence is mainly controlled by a Parallel Foils Collimator (PFC) and by the crystal analyzer; however, as it will be shown later by simulation, the major control over the divergence is played by the PFC (mainly equatorial divergence, reduced to tenths of a degree; the axial divergence is not greatly reduced, but limited to a few degrees) whereas the analyzer reduces the fluorescence signal, the axial divergence and cuts unwanted energies from the diffracted signal. In particular, the mosaicity of the flat crystal analyzer (pyrolitic graphite) contributes to limit the axial and equatorial divergence to a few hundredths of a degree (Gaussian distribution).

### 3.2 Beam Shape, Homogeneity, and Uniformity

The shape and uniformity of the beam reaching the specimen strongly depends on the properties of lens and x-ray source. The beam emerging from the focal spot of a sealed tube at a typical takeoff angle of 6° exhibits a non-circular shape, with local intensity maxima evenly distributed throughout the cross section, and shows a strongly divergent character. Moreover, it is well known from the literature, and experimentally observable, that an odd projection of the shape of the anode on the specimen produces unwanted features on peak tails (the so-called tube tails [23–24])

**Fig. 3:**
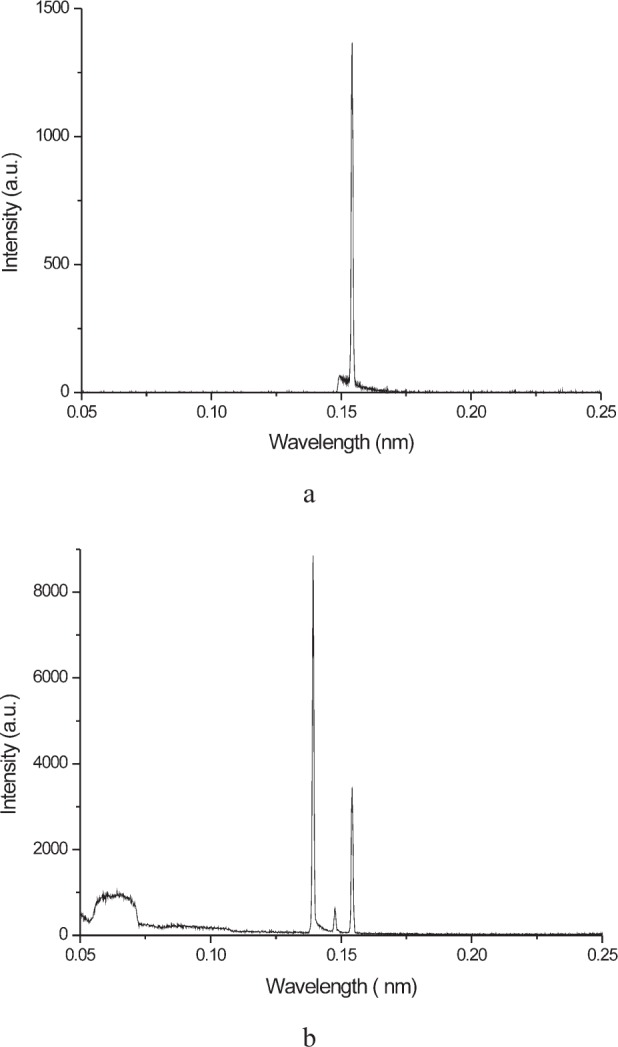
LiF patterns collected on the MRD I system in front of the lens using a 200 µm Cu filter at (a) 15 kV/15 mA and (b) 45 kV/40 mA. The wavelength has been calculated from the cell parameter of LiF, *a*_0_ = 0.4027 nm (ICDD-JCPDS PC-PDF card #04-0857).

The lens is expected to stop all energies higher than 10 keV, thus the spectral response of the tube-lens system should be improved over that of a traditional pinhole system. An effective way to picture the actual tube emission spectrum consists in the collection of the *θ*/2*θ* pattern of a LiF single crystal, as shown in Fig. 3 for MRD I. During data collection, the energy band-pass filter of the detector was totally open. Whereas at 15 kV only the K*_α_* doublet is visible, at 45 kV as set of extra features appear, namely the Cu-K*_β_* emission line at 0.139 nm and the W-L*_α_* line. The latter is due to tungsten contamination of the copper anode due to evaporation from the filament. The odd intensity ratio observed for the K*_α_* and K*_β_* lines (against an expected value of about 4) is due to the larger attenuation of the main spectral component by the copper foil used to shield the detector (cf. mass/absorption values corresponding to the two spectral components; as a reference [25]). The high energy signal (below ca. 0.12 nm) could be due to the electronic noise. The cut wavelength of the lens (about 0.12 nm) is therefore too low to stop some of the spurious signal present in the emission spectrum of the tube and a real gain in spectral purity cannot be inferred.

**Fig. 4:**
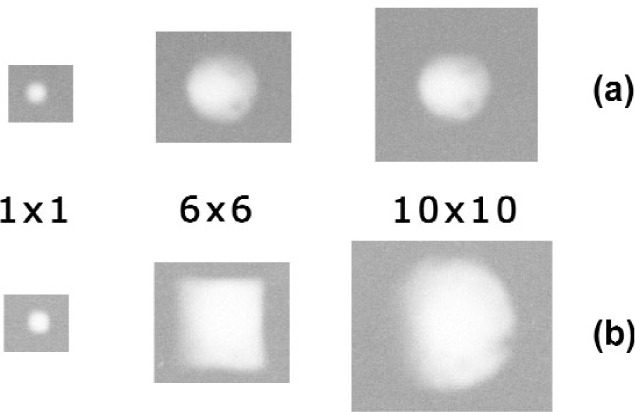
Polycapillary exit beam images of the (a) MRD I and (b) MRD II systems for a 1 × 1 mm, 6 × 6 mm, and 10 × 10 mm crossed slits aperture.

An additional feature, seldom considered for a pinhole or a crossed-slit system, is the distribution of the signal intensity across the section of the primary beam, expected to be a constant for an ideal instrument. This distribution could be directly imaged by means of a 2-D detector; however, should an area detector be unavailable, a (high resolution) x-ray film can be used to obtain a picture of the beam. With a flatbed scanner (the line scanner typically used for the analysis of x-ray films does not permit to collect area scans), the complete reconstruction of the intensity profile of the primary beam is then possible^2^. Figure 4 shows the result for both MRD I and MRD II at three different slit openings, namely 1 × 1 mm, 6 × 6 mm, and 10 × 10 mm. In all three cases the intensity distribution is nearly-Gaussian (as can be observed by fitting of a line scan taken through the film). The film was placed in the goniometer center (sample position), perpendicular to the lens and exposed for 2 min to a beam produced at 15 kV and 15 mA and filtered by a nickel foil (125 µm thickness). Besides the voltage/current difference, the emission spectrum is close to that present at the exit of the monochromator in normal operating conditions (cf. Fig. 3).

Missing-intensity spots are clearly visible for both lenses, and the intensity tends to decrease towards the outer lens circumference (Fig. 4). Possible explanation is the obstruction of some of the capillaries, e.g., by glass debris. The uneven intensity distribution along the lens radius induces a non linearity in the transfer function of the lens-slit assembly; in other words, the integrated intensity of the primary beam does not follow the increase of the area selected by the cross-slits collimator.

**Fig. 5:**
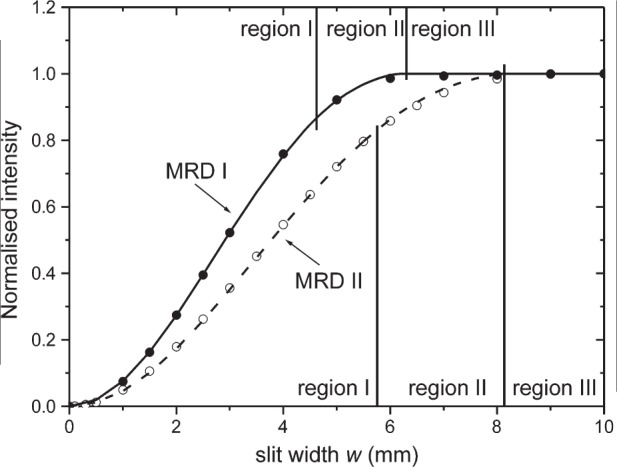
Intensity distribution for the MRD I (full dots correspond to raw data; line to model) and MRD II (open dots and dashed line) lenses recorded by varying the aperture in both directions.

To measure the transfer function indirectly, a detector is mounted in front of the collimator and the aperture of the two crossed slits is varied; the result is shown in Fig. 5 for MRD I and MRD II and provides an integrated information. The transfer function can be modeled both for the ideal case, always considered in the literature (uniform incoming beam), and for an incoming beam possessing the observed characteristics. A fit of the resulting equation to the measured data gives the parameters of the lens to be used for the Monte Carlo ray-tracing of the diffraction system.

#### Calculation in the Ideal Case

In an ideal case (uniform intensity, circular cross-section of the beam and crossed slits in front of it), three distinct regions can be identified, delimited by particular values of the slits aperture; they are marked with roman numerals in Fig. 5. A simple treatment of this case follows by using a square opening for the slits (horizontal and vertical dimensions of the primary beam are therefore equal to *w*) and supposing beam, polycapillary collimator and crossed-slits setup being concentric. The maximum for *w* will be indicated as *w*_max_. We can define an adimensional slit aperture $\hat{w}=w/(2R)$ where *R* is the nominal (i.e., outer) radius of the collimator (lens).

In *region I* the cross section of the beam is fully embedded in that of the collimator, i.e., the slit aperture is smaller than the edge of the largest square that can be inscribed in the lens circumference (the limiting slit aperture is thus $w_{I}=\sqrt{2R}$, i.e., ${\hat{w}}_{I}=\sqrt{2}/2$). In this region, the normalized transmitted intensity follows a parabolic law:

$$T_{I}(\hat{w})=\frac{4}{\pi }{\hat{w}}^{2}.$$ (1)

For bigger openings and up to the limit when the whole lens is exposed (i.e., in the range $\sqrt{2}/2\leq \hat{w}\leq 1$, *region II*), the normalized transmitted intensity can be expressed as:

$$T_{II}(\hat{w})=1+\frac{4}{\text{π}}\left(\hat{w}\sqrt{1-{\hat{w}}^{2}}-\text{arccos}(\hat{w})\right)$$ (2)

For openings bigger than 2*R* (*region III*), no variation of the transmitted beam is expected, as the entire lens is exposed $\left[T_{\mathrm{III}}\left(\hat{w}\right)=1]\right)$.

#### Calculation for the Non-Ideal Case

The non-uniformity of the primary beam can be introduced via a function describing the distribution of intensity through the section of the beam. Symmetry considerations suggest a function dependent exclusively on the position along the radius of the lens (Radial Intensity Distribution Function, RIDF). Following the previous observation of the direct beam (cf. Fig. 4) and the integrated measurement of Fig. 5, a Gaussian RIDF can be used:

$$I(\rho ,\sigma ,R)=I_{0}\frac{\text{ln}2}{\pi \sigma^{2}}\frac{\text{Exp}\left(-\frac{\rho^{2}}{\sigma^{2}}\text{ln}2\right)}{1-\text{Exp}\left(-\frac{R^{2}}{\sigma^{2}}\text{ln}2\right)}$$ (3)

where *I*(*ρ*, *σ*, *R*) is the intensity transmitted by an infinitesimal area at a distance *ρ* from the center of the polycapillary collimator, *I*_0_ is the total intensity transferred by the lens (i.e., the intensity measurable when the collimator slits are removed), *σ* is the Half Width at Half Maximum (HWHM) of the RIDF and *R* is the (outer) radius of the lens. This is, to some extent, a simplification of the problem (the actual picture is more complex as clear from Fig. 4). The functional form of Eq. (3), however, does not affect the treatment of the problem that preserves its generality.

Due to the crossed slits, only part of the intensity, namely the integral of the RIDF over the cross section of the beam, reaches the specimen. The more general case of a rectangular beam of width *w* and height *h* will be considered; as for the ideal case, the intensity transmission depends on the slits opening. *Region I* fulfils the requirement that the selected area lies entirely within the capillary boundary (i.e., *w*^2^ + *h*^2^ ≤ 4*R*^2^). The corresponding normalized transmitted intensity is^3^:

$$\begin{array}{c} T_{I}(w,h,\sigma ,R)=4\int_{0}^{h/2}\int_{0}^{w/2}\frac{I[\rho (x,y),\sigma ,R]}{I_{0}}dxdy\\ \;=\frac{\text{Erf}\left(\frac{h}{2}\frac{\sqrt{\ln2}}{\sigma }\right)\text{Erf}\left(\frac{w}{2}\frac{\sqrt{\ln2}}{\sigma }\right)}{1-\text{Exp}\left(-\frac{R^{2}}{\sigma^{2}}\ln2\right)} \end{array}$$ (4)

When the condition for *region I* is violated, but the horizontal and/or vertical dimensions of the beam are both less than 2*R*, we enter *region II*; an analytical solution for this case cannot be found and the result has to be computed numerically from:

$$\begin{array}{l} T_{II}(w,h,\sigma ,R)=1-\frac{2}{\pi }[\arccos(\hat{w})+\arccos(\hat{h})]\\ +\frac{4}{1-\text{Exp}\left(-\frac{R^{2}}{\sigma^{2}}\ln2\right)}\\ \left(\begin{array}{l} \int_{0}^{w/2}\text{Exp}\left(-\frac{x^{2}}{\sigma^{2}}\ln2\right)\text{Erf}\left(x\frac{\sqrt{\ln2}}{\sigma }\sqrt{{\hat{w}}^{-2}-1}\right)dx\\ +\int_{0}^{h/2}\text{Exp}\left(-\frac{x^{2}}{\sigma^{2}}\ln2\right)\text{Erf}\left(x\frac{\sqrt{\ln2}}{\sigma }\sqrt{{\hat{h}}^{-2}-1}\right)dx \end{array}\right) \end{array}$$ (5)

When only one of the two dimensions of the beam is bigger than 2*R*, *region III* is reached; in this region, the intensity follows directly from Eq. (5), provided that the dimension exceeding this limit is replaced by 2*R*. The solution *T_IV_*(*w*,*h*,*σ*, *R*) = 1 holds for *region IV*, i.e., when the lens is fully exposed.

It is worth noting that whenever the condition *w* = *h* is met, the four regions reduces to three as in the ideal case described before.

### 3.2 Model Testing

To assess the validity of the proposed solution, Eqs. (4) and (5) were fit to the experimental data of Fig. 5 by means of the commercial software package Origin Pro ver. 6.1 (Origin Labs inc.). The parameters obtained from the fit are reported in Table 1.

**Fig. 6:**
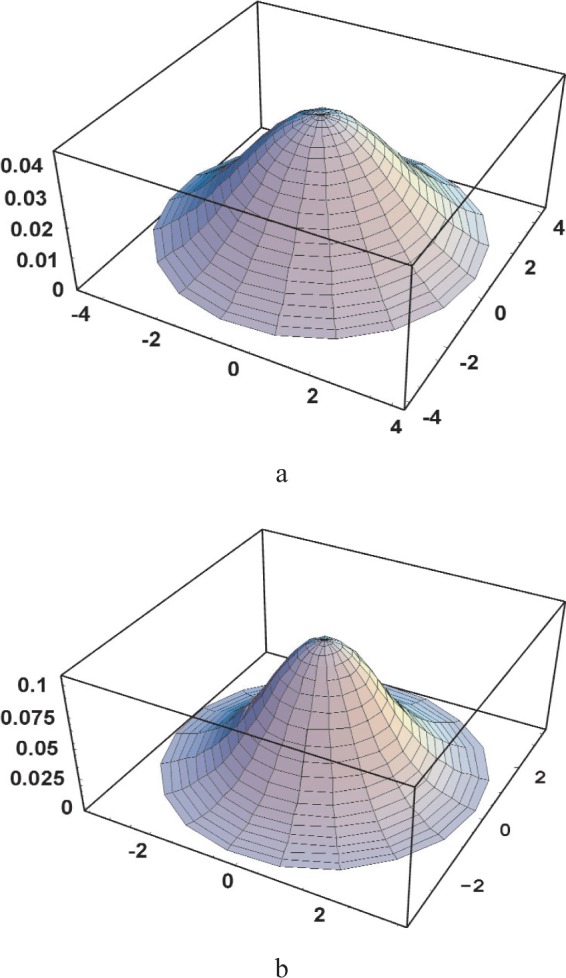
Modelling of the radial intensity distribution for the (a) MRD I and (b) MRD II lenses.

An effective radius, lower than the geometrical dimension of the lens assembly (nominal radius) is obtained (edge effects are therefore present). Moreover, different widths of the intensity distribution were obtained for the various lenses (cf. Fig. 6a and b). There are different interpretations of this behavior, all due to the non-ideal nature of the lens. In any case, as also suggested by the instrument manufacturer, the maximum size of the beam should be limited to few millimeters in both directions to guarantee an optimal response of the instrument.

**Fig. 7:**
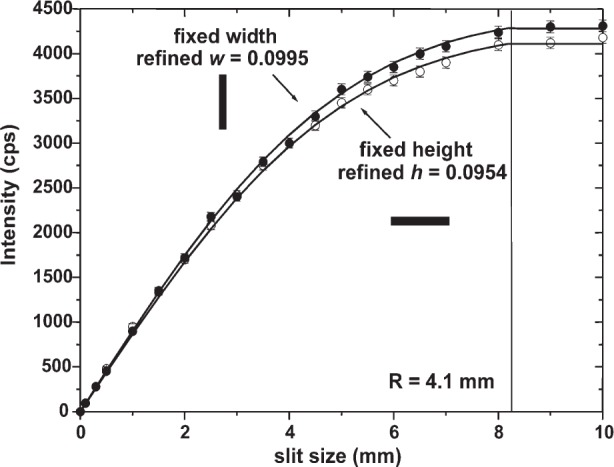
Intensity distribution for the MRD II lens recorded by varying the aperture independently in the vertical (fixed width, full dots) and horizontal (fixed height, open dots) directions. The nominal fixed height (width) was 0.1 mm. Modelling results for both cases are shown as continuous line (fixed width) and dashed line (fixed height), respectively.

An additional set of intensity measurements (average over 10 s, 10 µm Cu attenuator used) was conducted at various slit openings by keeping one of the dimensions of the beam fixed to a nominal value of 0.1 mm. The result for MRD II is shown in Fig. 7; the two sets of experimental points represent the integral of the radial distribution function performed along two perpendicular directions (*w* and *h* is varied, respectively). For a narrow slit, the integrated intensity (without normalization) can be written with a good approximation as

$$I_{\text{narrow}}(h,w,\rho ,R)=I_{0}\cdot T_{I}(h,w,\sigma ,R)$$ (6)

where either *h* or *w* (or both) must be small and where *I*_0_ still represents the intensity measurable when the lens is fully exposed. The formula is valid up to an aperture of the slits equal to the diameter of the lens (for bigger apertures, being the lens fully exposed, the intensity remains constant at *I*_0_).

The data previously obtained (Table 1) were inserted in Eq. (6) to reproduce the trend of Fig. 7; moreover *I*_0_ (194 000 cps) was obtained from an intensity measurement conducted at maximum aperture (10 mm × 10 mm) whereas the value of the fixed dimension, i.e., *h* (respectively, *w*) was refined. The difference between the two curves can in fact be attributed to a slight error in the position of the slit that was kept fixed; in particular *w* = 0.0978 mm and *h* = 0.0947 mm (expected values 0.1 mm) were refined in the two cases, respectively (see Table 2). The slits were positioned manually, thus the given explanation is fully justified.

The agreement between data and model confirms that the chosen RIDF well reproduces the features of the lens even when some degree of non-homogeneity is present.

## 4. Monte Carlo Raytracing of the System

The measured profile *h* can be obtained as convolution of the sample broadening effects *f* with the instrumental profile *g* (i.e., *h* = *f* ⊗ *g*). The separation of the various contributions is still a hot topic in the literature and both convolutive and deconvolutive approaches have been proposed and tested. Among them, the Fourier deconvolutive approach is probably the most frequently applied to date as it combines calculation speed with a physical significance of the results (in the Fourier formulation, the convolution integral is transformed in a product, greatly simplifying the mathematical complexity of the problem).

In the deconvolutive approach, the instrumental contribution is unfolded from the measured profile and the whole analysis is performed on the extracted *f* function, thus replacing the original raw data with the deconvolved data. Convolutive approaches instead, work directly on the measured data, building the expected *h* profile from a model description of the *f* and *g* functions. In this way, parameters referring both to the specimen and to the instrument can be refined together by modelling the measured data. The so-called Fundamental Parameters Approach (FPA; for details see, e.g., Ref. [23,24,26–31]), i.e., the analytical modeling of the instrumental profile from the physical dimensions of the optical devices present in the diffractometer, can also be used.

With respect to the deconvolutive approaches, convolutive methods preserve the original (raw) data and the associated statistics, resulting in a higher accuracy and physical significance of the results. In the following, the convolutive route will be thus followed.

The FPA has been recently proposed in a fully analytical version for the determination of the *θ*/2*θ* diffraction patterns both for laboratory instruments (see, e.g., Refs. [23,24,27–33]) and for large-scale facilities (neutron diffraction and synchrotron radiation x-ray diffraction). In all cases, the modeling was possible because of the simple nature of the problem. More complex problems (e.g., non-conventional optical components or complex systems) can be modeled by Monte Carlo ray-tracing: as an example, see the SHADOW [34–35] or XOP [36–37] packages commonly used for the simulation of the x-ray response of complex optical devices.

In the proposed Monte Carlo raytracing, the path of a generic x-ray is calculated analytically from the source to the detector. Each optical device is modeled and its effect evaluated for a single ray (spatial/angular filtering). A set of random rays is generated, possessing the characteristics (intensity/divergence) known for the primary beam, and their path followed from the source to the detector (if the latter is reached).

The non-uniform intensity distribution in the primary beam and complex movements of the specimen can be thus considered. The flexibility is paid in terms of efficiency, the calculation speed being orders of magnitude lower than for a correspondent fully analytical case (as in Refs. [27–30,32], for instance).

One of the features of the Monte Carlo algorithm is the asymptotic convergence. There is a critical number of rays above which increasing the number of rays does not appreciably increases the accuracy of the result. For our case, the critical value is about 5 × 10^6^ rays.

Since the raytracing procedure computes only the instrumental effects, both the emission profile and the sample broadening contribution (supposed to be absent, thus modeled by a Dirac’s delta function) must be given. For the emission profile, the data for copper radiation given by Hölzer et al. [22] is used.

To reduce the complexity of the raytracing, a set of suitable reference systems will be considered, as in Fig. 1. Each coordinate is given a superscript indicating the reference frame in which it is considered. Whereas italic non-bold letters denote scalars (e.g., *v*), an arrow is used to identify a vector (e.g., $\vec{v}$) and a hat to mark a unit vector (e.g., $\hat{v}=\vec{v}/v$). Following this convention, a vector represented in the reference frame B is identified as ${\vec{v}}^{B}=(x^{B},y^{B},z^{B})$. Unless otherwise specified, rotations are counterclockwise.

### 4.1 Primary Beam: X-Ray Lens and Specimen

The reference systems used throughout the text are reported in Fig. 1 whereas Table 3 summarizes the main parameters describing the diffraction system. The reference system **G** fixed in the laboratory has the origin in the goniometric center^4^ (see Refs. [28,29]), the *y* axis pointing towards the source when *θ* = 0 and the *x* axis normal to the surface of the specimen when *ψ* = 0 (for the definition of the angles, see Fig. 1).

Let us consider the primary ray $\vec{r}=\vec{P}+\xi \hat{D}$ originating in $\vec{P}$ and directed along $\hat{D}$ (ξ is the running coordinate, i.e., the norm of the distance between $\vec{r}$ and $\vec{P}$). In the reference frame **L**, whose *x* and *y* axes lie on the cross section of the polycapillary collimator, the point $\vec{P}$ is represented as ${\vec{P}}^{L}=(x_{0}^{L},y_{0}^{L},0)$. The vector $\hat{D}$ carries the information about the divergence of the ray; it can be easily constructed as to have an axial divergence Δ*α* and an equatorial divergence Δ*θ* by rotating the vector (0, 1, 0) in **G** about the *x* axis by the angle Δ*α* and subsequently about the *z* axis by the angle *θ* + Δ*θ* (rotation matrices ***R***_3_ and ***R***_4_, respectively):

$$\begin{array}{l} R_{3}=\left(\begin{array}{ccc} 1 & 0 & 0\\ 0 & \cos(\Delta \alpha ) & \sin(\Delta \alpha )\\ 0 & -\sin(\Delta \alpha ) & \cos(\Delta \alpha ) \end{array}\right)\\ R_{4}=\left(\begin{array}{ccc} \cos(\theta +\Delta \theta ) & \sin(\theta +\Delta \theta ) & 0\\ -\sin(\theta +\Delta \theta ) & \cos(\theta +\Delta \theta ) & 0\\ 0 & 0 & 1 \end{array}\right) \end{array}$$ (7)

With these definitions, ${\hat{D}}^{G}=R_{4}\cdot R_{3}\cdot {\left(0,1,0\right)}^{T}$. Since the point *P* can be represented in **G** as:

$${\vec{P}}^{G}=\left(\begin{array}{c} x_{0}^{G}\\ y_{0}^{G}\\ z_{0}^{G} \end{array}\right)=\left(\begin{array}{c} -x_{0}^{L}\cos(\theta )+d_{11}\sin(\theta )\\ x_{0}^{L}\sin(\theta )+d_{11}\cos(\theta )\\ y_{0}^{L} \end{array}\right)$$ (8)

we can obtain the parametric equation for the primary ray

$${\vec{r}}^{G}=\left(\begin{array}{c} x^{G}\\ y^{G}\\ z^{G} \end{array}\right)={\vec{P}}^{G}+\xi {\hat{D}}^{G}=\left(\begin{array}{c} x_{0}^{G}\\ y_{0}^{G}\\ z_{0}^{G} \end{array}\right)+\xi \left(\begin{array}{c} \cos(\Delta \alpha )\sin(\theta +\Delta \theta )\\ \cos(\Delta \alpha )\cos(\theta +\Delta \theta )\\ -\sin(\Delta \alpha ) \end{array}\right)$$ (9)

The surface of the specimen displaced by the quantity *δ* (so as to lay in the plane *x***^G^** = –*δ*) and rotated about the axis y of **G** by an angle *ψ* (specimen tilting), is described in **G** by:

$$x^{G}\cos(\psi )+z^{G}\sin(\psi )+\delta =0.$$ (10)

The common solution of Eqs. (9) and (10), i.e., the solution of the system Eq. (11):

$$\left(\begin{array}{cccc} 1 & 0 & 0 & -\cos(\Delta \alpha )\sin(\theta +\Delta \theta )\\ 0 & 1 & 0 & -\cos(\Delta \alpha )\cos(\theta +\Delta \theta )\\ 0 & 0 & 1 & \sin(\Delta \alpha )\\ \cos(\psi ) & 0 & \sin(\psi ) & 0 \end{array}\right)(\begin{array}{c} x_{h}^{G}\\ y_{h}^{G}\\ z_{h}^{G}\\ \xi_{h} \end{array})=\left(\begin{array}{c} x_{0}^{G}\\ y_{0}^{G}\\ z_{0}^{G}\\ -\delta \end{array}\right)$$ (11)

gives the point ${\vec{H}}^{G}=(x_{h}^{G},y_{h}^{G},z_{h}^{G})$ of intersection of $\vec{r}$ with the surface of the specimen (ξ_h_ is the distance between $\vec{P}$ and $\vec{H}$). In the reference **S** aligned with the sides of the specimen, the coordinates of the hit point become:

$$\left(\begin{array}{c} x_{h}^{S}\\ y_{h}^{S} \end{array}\right)=\left(\begin{array}{c} y_{h}^{G}\\ -x_{h}^{G}\sin(\psi )+z_{h}^{G}\cos(\psi ) \end{array}\right)$$ (12)

A quicker way to consider the rotation of the specimen about its normal, is to perform a counter-clockwise rotation of the coordinates $x_{h}^{S}$, $y_{h}^{S}$ by the angle *ϕ* (to represent the hit point in the reference system centered on the specimen and aligned with the sides of it, cf. Fig. 1):

$$\left(\begin{array}{c} x_{h}^{S,\text{true}}\\ y_{h}^{S,\text{true}} \end{array}\right)=\left(\begin{array}{c} x_{h}^{S}\cos(\phi )-x_{h}^{S}\sin(\phi )\\ x_{h}^{S}\sin(\phi )+y_{h}^{S}\cos(\phi ) \end{array}\right)$$ (13)

(the specimen is supposed to be rotated clockwise by *ϕ*).

When the conditions $\left|SW\right|\leq 2x_{h}^{S,\text{true}}$ and $\left|SW\right|\leq 2\;y_{h}^{S,\text{true}}$ are satisfied, diffraction occurs; if not, the tracing of the ray will end (the treatment is valid whatever the shape of the specimen. If we call *Σ* the surface of the specimen, the condition transforms into $\left(x_{h}^{S,\text{true}},y_{h}^{S,\text{true}})\in \Sigma \right)$.

### 4.2 Secondary Beam: Parallel Foils Collimator and Crystal Analyzer

In the raytracing calculation, every ray hitting the surface of the specimen generates a diffraction cone. In a real measurement, this is not necessarily the case: the occurrence of diffraction is a statistical event and most incoming rays are not diffracted but absorbed in the specimen. However, this effect need not be considered as the number of rays reaching the detector would just be scaled by a constant factor. An analogous reasoning is valid for texture, simply changing the partition of intensity along different scattering directions (texture can therefore be considered *a posteriori*).

For the diffractometers analyzed here, the source is fixed and both specimen and source rotate about the goniometric axis; however, a simpler mathematical model can be obtained if the opposite is done, i.e., if specimen is considered stationary and both source and detector rotate. These two operation modes are equivalent.

To simulate the diffraction event and the collection of the diffracted signal, we should consider both the diffraction (Bragg) angle *θ*_B_ and the detector angle *θ*_d_, the first dependent on the material (interplanar spacing), the second imposed by the scan mode (that establishes the direction along which the secondary arm is positioned).

A cone $\vec{d}\left(\xi ,\chi \right)$ is defined by the parametric equation:

$$\begin{array}{l} \vec{d}(\zeta ,\chi )=\left(\begin{array}{c} x\\ y\\ z \end{array}\right)=\vec{P}+\xi \hat{E}(\chi )\\ \;=\vec{P}+\xi \left[\vec{U}+\vec{V}\cdot \cos(\chi )+\vec{W}\cdot \sin(\chi )\right]\\ \;=\left(\begin{array}{c} x_{0}\\ y_{0}\\ z_{0} \end{array}\right)+\xi \left[\left(\begin{array}{c} u_{1}\\ u_{2}\\ u_{3} \end{array}\right)+\left(\begin{array}{c} v_{1}\\ v_{2}\\ v_{3} \end{array}\right)\cos(\chi )+\left(\begin{array}{c} w_{1}\\ w_{2}\\ w_{3} \end{array}\right)\sin(\chi )\right] \end{array}$$ (14)

where $\vec{P}$ is the *vertex* of the cone (in our case the point where diffraction occurs, i.e., $\vec{H}$) and $\hat{E}(\chi )$ is the *directrix* (i.e., in this case, the set of all diffracted rays, parameterized by the rotation angle about the axis of the cone). The directrix can be conveniently decomposed along three orthogonal vectors $\vec{U}$ $\vec{V}$ $\vec{W}$ with $\vec{U}$ as the axis of the cone.

To generate the diffraction cone relative to $\hat{D}$, we can start from the ray (0,1,0) in **G** generating the corresponding diffraction cone with the vertex in the origin of the axes. Subsequently, the rotations described previously [cf. Eq. (7)] produce the expected set of rays. With a proper selection of the sequence, only rotations around the axes of **G** need be employed. Starting from (0,1,0) a clockwise rotation by *π* – 2*θ*_b_ about the *z* axis (rotation matrix ***R***_1_) gives the directrix of the diffraction cone

$$\begin{array}{l} R_{1}=\left(\begin{array}{ccc} \cos\left(\pi -2\theta_{b}\right) & \sin\left(\pi -2\theta_{b}\right) & 0\\ -\sin\left(\pi -2\theta_{b}\right) & \cos\left(\pi -2\theta_{b}\right) & 0\\ 0 & 0 & 1 \end{array}\right)\\ \;=\left(\begin{array}{ccc} -\cos\left(2\theta_{b}\right) & \sin\left(2\theta_{b}\right) & 0\\ -\sin\left(2\theta_{b}\right) & -\cos\left(2\theta_{b}\right) & 0\\ 0 & 0 & 1 \end{array}\right) \end{array}$$ (15)

Consequently, the diffraction cone can be obtained by rotating the directrix about the *y* axis (matrix ***R***_2_)

$$R_{2}=\left(\begin{array}{ccc} \cos\left(\chi \right) & 0 & -\sin\left(\chi \right)\\ 0 & 1 & 0\\ \sin\left(\chi \right) & 0 & \cos\left(\chi \right) \end{array}\right)$$ (16)

and is characterized by the equation *ξ R*_2_ · *R*_1_ · (0,1,0)*^T^*. It is worth noting that the direction of rotation in ***R***_2_ does not play any role in the problem since a complete revolution is needed to generate the whole cone. The cone has then to be rotated in order to align its axis to the primary ray $\hat{D}$, by means of the matrices [Eq. (7)]. Since the cone is centered in the origin of **G**, the rotation would affect only the directrix.

In order to understand whether the ray will reach the detector or will be filtered by the optical devices positioned along the secondary path, we look for the intersection of the diffraction cone with the parallel plates collimator and with the analyzer. The calculation will be conveniently conducted in **D**, obtained by the clockwise rotation of **G** about *z* by the angle π−*θ*_d_. In this way, the *y* axis of the **D** frame is aligned along the secondary arm, whereas the entrance and exit sections of the parallel foils collimator lie on two parallel planes whose equations are *y*^D^ = *d*_21_ and *y*^D^ = *d*_21_ + *d*_22_, respectively. At this point, a rotation about the *z* axis of **G** suffice to move all the problem in the reference system **D**. The coordinate transformation matrix $R_{G}^{D}$ to be used is:

$$\begin{array}{l} R_{G}^{D}=\left(\begin{array}{ccc} \cos\left(\pi -\theta_{d}\right) & -\sin\left(\pi -\theta_{d}\right) & 0\\ \sin\left(\pi -\theta_{d}\right) & \cos\left(\pi -\theta_{d}\right) & 0\\ 0 & 0 & 1 \end{array}\right)\\ \;=\left(\begin{array}{ccc} -\cos\left(\theta_{d}\right) & -\sin\left(\theta_{d}\right) & 0\\ \sin\left(\theta_{d}\right) & -\cos\left(\theta_{d}\right) & 0\\ 0 & 0 & 1 \end{array}\right) \end{array}$$ (17)

(note the discordance of signs with respect to ***R***_4_. Consider that Eq. (17) transforms a frame, i.e., changes the reference, whereas ***R***_4_ rotates a vector in a fixed frame).

The final expression for the diffraction cone (represented in the system **D**) is obtained by combination of the matrices:

$$\begin{array}{l} \left(\begin{array}{c} x^{D}\\ y^{D}\\ z^{D} \end{array}\right)=R_{G}^{D}\left[{\vec{P}}^{G}+\xi {\hat{E}}^{G}\left(\chi \right)\right]\\ \;=R_{G}^{D}[\left(\begin{array}{c} x_{h}^{G}\\ y_{h}^{G}\\ z_{h}^{G} \end{array}\right)+\xi R_{4}\cdot R_{3}\cdot R_{2}\cdot R_{1}\cdot \left(\begin{array}{c} 0\\ 1\\ 0 \end{array})\right]. \end{array}$$ (18)

The point $\left[x_{c}^{D}\left(\kappa \right),\kappa ,z_{c}^{D}\left(\kappa \right)\right]$ determined by the intersection of the diffracted beam with the plane *y***^D^** = *κ* in the secondary path, obeys the following system of equations:

$$\left(\begin{array}{c} x^{D}\\ y^{D}\\ z^{D} \end{array}\right)=R_{G}^{D}\left[\left(\begin{array}{c} x_{h}^{G}\\ y_{h}^{G}\\ z_{h}^{G} \end{array}\right)+\xi R_{4}\cdot R_{3}\cdot R_{2}\cdot R_{1}\cdot \left(\begin{array}{c} 0\\ 1\\ 0 \end{array}\right)\right]=\left(\begin{array}{c} x_{c}^{D}\left(\kappa \right)\\ \kappa \\ z_{c}^{D}\left(\kappa \right) \end{array}\right)$$ (19)

It is worth noting that due to our choices, the components of the directrix of the cone ${\hat{E}}^{G}\left(\chi \right)$ are the tangent of the angles formed by the diffracted ray and the axes of **D**. In particular, the *z* and *x* components are the divergence angles of the diffracted beam in the axial and equatorial direction, respectively, with respect to the reference **D**.

The solution of Eq. (19) can be used to evaluate the position of the ray both at the entrance and exit section of the parallel foils collimator. If $\left|CW\right|\leq 2x_{c}^{D}\left(d_{21}+d_{22}\right)$ and $\left|CH\right|\leq 2z_{c}^{D}\left(d_{21}+d_{22}\right)$ then the beam will be analyzed by the parallel foils collimator (the condition that it could actually exit the collimator is evaluated). The PFC has a multiple effect: it limits the equatorial divergence (2*θ_d_*_max_ = *CW*/*d*_22_), it cuts part of the beam (finite entrance section) and it limits the axial divergence (2*α*_max_ = *CH*/*d*_22_). The axial and equatorial divergence to be considered are those derived from the directrix of the cone.

The number of parallel foils in the collimator should be also considered, lowering the number of rays that reaches the analyzer (masking effect). From the practical point of view, each couple of parallel foils of length *d* and spacing *s* is equivalent to a set of narrow slits of angular aperture arctan(*d*/*s*); their filtering effect depends not only on the incidence angle of the beam, but also on the relative position of the beam with respect to the entrance section (a beam very close to one of the foils is not totally equivalent to a ray arriving in the middle between two foils). The intensity of the beam exiting the collimator has to be corrected for this effect: to this purpose we use a triangular function (as convolution of the box functions describing the entrance and exit slits composing a parallel foils pair). According to the horizontal position $x_{c}^{D}\left(d_{21}+d_{22}\right)$ on the exit section of the PFC $\left(0\leq x_{c}^{D}\left(d_{21}+d_{22}\right)\leq CW/2\right)$, the intensity of the ray is scaled by a factor $\left(1-2x_{c}^{D}\left(d_{21}+d_{22}\right)/CW\right)$.

The intersection of the beam with the plane of the analyzer follows the case of the collimator:

$$\left(\begin{array}{c} x^{D}\\ y^{D}\\ z^{D} \end{array}\right)=\left(\begin{array}{c} x_{a}^{A}\sin\left(\theta_{a}\right)\\ d_{a}-x_{a}^{A}\cos\left(\theta_{a}\right)\\ y_{a}^{A} \end{array}\right)$$ (20)

where $x_{a}^{A}$ and $y_{a}^{A}$ are the coordinates of the intersection point expressed in the reference system **A** aligned with the edges of the analyzer, *d*_a_ is the distance of the center of the analyzer to the origin of **G** (*d*_a_ = *d*_21_ + *d*_22_ + *d*_23_) and *θ*_a_ is the angle of the analyzer with respect to the secondary arm (2*θ*_a_ = 26.57° for a (002)-graphite crystal).

Lying on a plane perpendicular to the equatorial plane, the analyzer can be considered (from a geometrical point of view) as a mirror for the x-rays, thus actual calculations of the reflection follow easily from Eq. (19). The position on the detector is thus (−*y*_c_(*d*_21_ + *d*_22_ + *d*_23_ + *d*_24_), *z*_c_(*d*_21_ + *d*_22_ + *d*_23_ + + *d*_24_)).

The ray is detected if $\sqrt{\left(y_{c}{\left(d_{21}+d_{22}+d_{23}+d_{24}\right)}^{2}+z_{c}{\left(d_{21}+d_{22}+d_{23}+d_{24}\right)}^{2}\right)}\leq RD$, *RD* being the radius of the sensitive area of the detector.

### 4.3 Integrated Intensity

The proposed scheme allows the calculation of the path for all possible rays leaving the polycapillary collimator. To obtain a diffraction profile, i.e., to actually mimic the diffraction experiment, an intensity value has to be attributed to each ray, dependent on the characteristics of the capillary and of the optical devices crossed by the ray. The contribution of the ray $\vec{r}$ exiting from the lens at a distance *ρ* from its center, can be written as:

I=I(ρ,σ,R)⋅F⋅T.

The various contributing terms are as follows:

- *I*(*ρ*,*σ*, *R*) describes the (uneven) intensity distribution on the exit section of the lens. The polar coordinate *ρ* (distance from the center of the lens) can be replaced by Cartesian coordinates, leading to:
  $$I\left(\rho ,x_{p},y_{p},R\right)=I_{\text{max}}\pi R^{2}\frac{\ln2}{\pi \sigma^{2}}\frac{\exp\left(-\frac{x_{p}^{2}+y_{p}^{2}}{\sigma^{2}}\ln2\right)}{1-\exp\left(-\frac{R^{2}}{\sigma^{2}}\ln2\right)}$$
  where *I*_max_ is the maximum intensity, measurable in the middle of the lens.
- *F* accounts for the distribution of divergence angles in the primary beam. Following the discussion in Sec. 3.1, we can consider a Gaussian distribution of angles: $F=\frac{\sqrt{\ln2}}{\sigma_{\beta }\sqrt{\pi }}\exp\left(-{\left(\frac{\Delta \beta }{\sigma_{\beta }}\right)}^{2}\ln2\right)$. expression Δ*β* is the angle between a given ray and an ideal ray possessing no divergence, whereas *σβ* is the HWHM of the distribution curve (≈0.3°, cf. Sec. 3.1). The angle *β* can be calculated from the known Δ*α* and Δ*θ* angles as Δ*β* = arccos[cos(Δ*α*)cos(Δ*θ*)].
- *T* accounts for absorption and (possible) thin film effects. If the specimen is a powder layer of finite thickness (or, equivalently, a randomly-oriented thin film), indicating by *t* the thickness of the layer, by *τ* the *information depth* [17] and by *µ* the linear absorption coefficient, *T* reads:
  $$T=\frac{1-\exp\left(-t/\tau \right)}{2\mu }\cos\left(\psi \right).$$

For the *ψ*-tilt geometry considered here, the information depth is *τ* = sin(*θ*)cos(*ψ*)/(2*µ*).

The contribution of the ray $\vec{r}$ thus becomes:

$$\begin{array}{l} I=I\left(\rho ,\sigma ,R\right)\cdot F\cdot T\\ =I_{\max}\frac{1}{\sigma^{2}}{\left(\frac{\ln2}{\pi }\right)}^{3/2}\frac{\exp\left(-\frac{x_{p}^{2}+y_{p}^{2}}{\sigma^{2}}\ln2\right)}{1-\exp\left(-\frac{R^{2}}{\sigma^{2}}\ln2\right)}\exp\left(-{\left(\frac{\Delta \beta }{\sigma_{\beta }}\right)}^{2}\ln2\right)\\ \left[1-\exp\left(-\frac{2\mu t}{\sin\left(\theta \right)\cos\left(\psi \right)}\right)\right]\frac{\cos\left(\psi \right)}{2\mu }. \end{array}$$

Further effects (uniformly) reducing the intensity of each ray are the finite thickness of the foils (reduction of the total intensity by the ratio of the empty cross-section area to the total cross-section area) and the scattering/absorption of air (the complete path-length is known for each ray). For a complete evaluation, the transmittance functions of PFC and crystal analyzer, should be included as well. The effect of these parameters, however, is negligible (or just a scale factor) and therefore will not be considered in detail.

### 4.4 Calculation of the *θ*/2*θ* Pattern

The instrument-broadening profile can be obtained by fixing 2*θ*_b_ in correspondence to the expected peak maximum (Bragg angle), and then simulating a *θ*/2*θ* scan by taking a Dirac delta function to model specimen response. Following the experimental practice, the detector angle *θ*_d_ is set equal to the incident angle *θ*, and for each angle *θ* considered, a sufficient number of rays is generated with a divergence distributed according to Sec. 4.3 (to guarantee the convergence of the Monte-Carlo algorithm). The rays are traced and the thus obtained (cumulated) intensity, assigned to the 2*θ*_d_ angle. An ideal diffraction system would give a diffracted signal only when *θ* = *θ*_b_ = *θ*_d_; in our case, on the contrary, broadened peaks due to the geometrical features of the instrument are obtained.

The emission profile and the sample related (size/strain broadening) profile can then be convoluted to the instrument profile to obtain the expected diffraction line shape.

It is worth noting here that this is a simplified calculation scheme, giving reasonably good results. A more rigorous approach would consider a distribution of wavelengths in the Monte-Carlo raytracing (for instance following Hölzer et al. [22]): each ray would be assigned a wavelength according to the distribution, and a sufficient number of rays would be chosen in order to consider a proper number of wavelengths. This would increase considerably the calculation time, but would probably not provide any significant contribution to the quality of the results; the satisfactory agreement between simulations and measured profiles (cf. Sec. 5) supports this hypothesis.

To finally contribute to the measured intensity, a ray must cross all optical devices and reach the detector. To increase the computation speed, the sequence of evaluation of the position of the ray at the entrance (exit) of the various optical components must be conveniently chosen. Once the position and the direction of the diffracted ray is known, the position of the ray on the entrance section of the detector is first calculated [using Eq. (19)]. If the ray has a chance to hit the detector window, then the other optical devices, in inverse order, are considered (i.e., crystal analyzer and parallel foils collimator). In this way, only the rays that have chances to reach the detector are actually traced.

## 5. Simulation and Comparison With Experimental Data

The proposed raytracing algorithm can be conveniently integrated in a larger frame of model fitting or data processing. From this point of view, in order to preserve the statistical meaning of the measurements, the raytracing results (i.e., the transfer function for the instrument) should not be used to pre-process the raw data but as an active element in the model.

To avoid the introduction of interpretative models and for displaying/qualitative analysis only, in the following, the application of the correction curves to raw data will be shown. It should be stressed that the procedure is not wrong in that only the statistical significance of the result (i.e., the error associated to the extracted parameters) is affected.

### 5.1 Intensity Effects in Texture Analysis

A set of pole figures is traditionally used to obtain the orientation distribution function (ODF) for a given specimen (for more details on the topic see, e.g., Ref. [38]). However, just a small portion of a pole figure (inner core) can be directly employed without corrections: in fact, data collected by a parallel beam diffractometer at low *ψ* and high 2*θ* are marginally affected by instrumental (and specimen-related) aberrations.

**Fig. 8:**
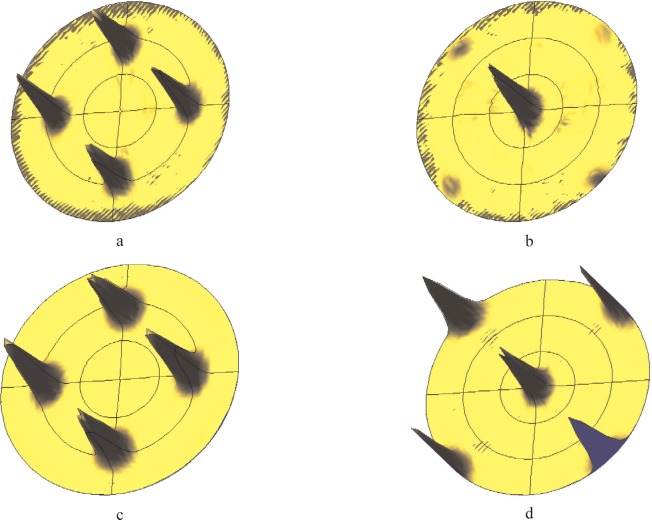
Highly textured ceria thin film. In (a) and (b) the (111) and (200) measured pole figures are shown. The corresponding simulations on the basis of the ODF calculated for the film from the inner core of a larger set of pole figures are shown in (c) and (d). Due to instrumental effects, the poles on the rim of (d) can hardly be seen in the actual measurement.

An experimental example will be used to clarify the problem: Figs. 8a and 8b show the 111 and 200 pole figures for a cerium oxide thin film produced by laser ablation [39]. Due to the particular texture of the film (cube on cube), two independent pole figures are sufficient for a full reconstruction of the orientation distribution function.

In particular, the ODF can be obtained by using only the inner core of the (111) and (200) pole figures (0° to 50° tilting), almost unaffected by instrumental aberrations. If the so-obtained ODF is used to reconstruct back the two generating pole figures (see Figs. 8c and 8d), features on the outer ring appear, not matched by the experimental data.

**Fig. 9:**
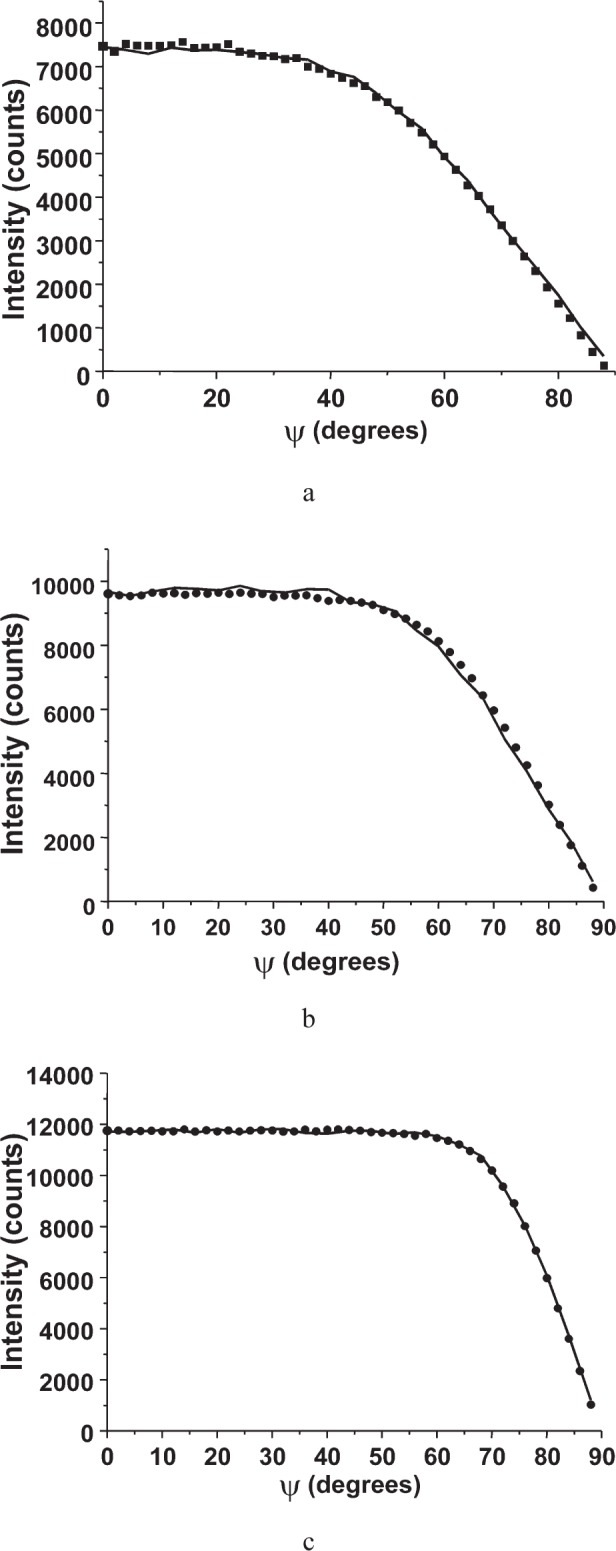
Tungsten powder: (a) low (b) mid and (c) high angle measurements. The reflections shown are (110) at 2*θ* = 40.26*°*, (220) at 2*θ* = 87.02°, and (321) at 2*θ* = 131.39°, respectively. The same geometrical parameters (MRD I) were used to simulate all curves. Full dots correspond to raw data, lines to the results of simulation.

**Fig. 10:**
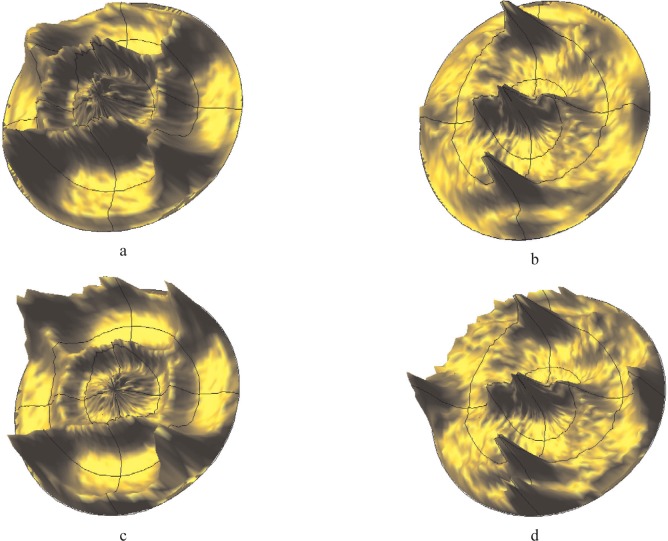
(a) measured (200) and (b) (220) pole figures for a Ni(V) specimen subject to cold rolling. The corresponding corrected data (expected pole figures) are shown in (c) and (d), respectively.

**Fig. 11:**
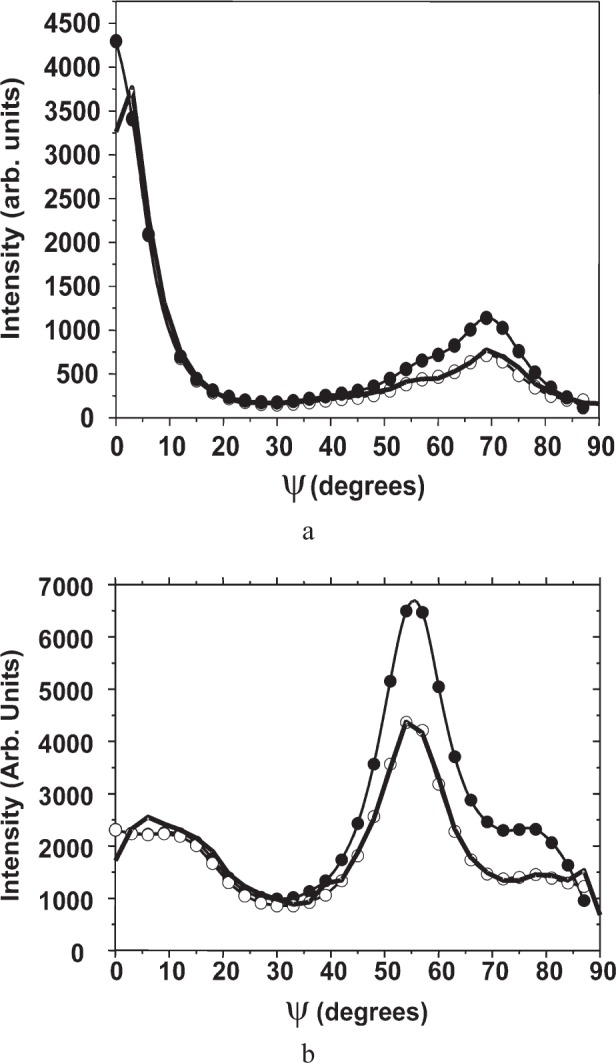
Sections through the (a) (111) and (b) (220) pole figures for a <111> fibre-textured thin copper film produced by magnetron sputtering: measured data (full dots), corrected data (open circles) and modelled curve (line). Measurement conducted on MRD I.

**Fig. 12:**
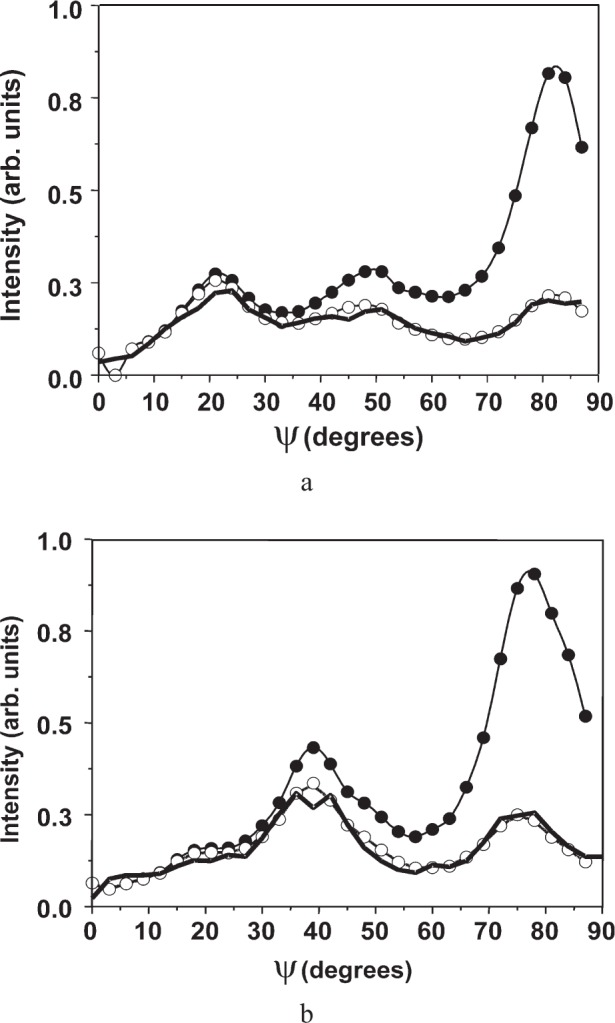
Sections through the (a) (331) and (b) (420) pole figures for a <111> fibre-textured thin copper film produced by magnetron sputtering: measured data (full dots), corrected data (open circles) and curves obtained by means of the ODF extracted from the (111) and (200) pole figures (line). Measurement conducted on MRD I.

Some additional examples are provided to show the performance of the raytracing procedure in real cases of study:

- Figure 9 shows the 110, 220, and 321 reflections of the tungsten powder (details can be found in Ref. [19]). Using the same instrumental parameters, the correction curves can be generated on a wide 2*θ* range. The agreement between data and model is excellent. The rippling in the curves is due to round-off errors in the calculations and statistical variations in the Monte Carlo algorithm.
- A particular texture is developed by a Ni(V) alloy subjected to cold rolling: (200) and (220) raw pole figures are shown in Figs. 10a and 10b, respectively, together with the corresponding corrected data (Figs. 10c and 10d, respectively). The effect of the correction on the outer rim of the pole figure is clearly visible; the measured intensity on the outer rim is quite low with respect to the expected value.
- Copper films produced by sputtering present a complex fiber texture. The {111} and {200} pole figures for a 500 nm film (data on the stress of these films have already been presented elsewhere; see for instance Ref. [20]) is shown in Fig. 11. These two pole figures can be corrected and then used by the commercial X’Pert Texture software to reconstruct the ODF for the film. From the ODF obtained in this way, the {331} and {420} pole figures can be simulated. The agreement between simulation and raw {331} and {420} pole figures (corrected using the raytraced data) is excellent, as shown in Fig. 12.

#### Correction for Specimen Rotation

In principle, even pole figures of a fiber-textured specimen (i.e., rotationally symmetric texture), can in some cases lose their symmetry because of an odd specimen shape. The case of rectangular specimens is a typical example; the raytracing can then be used to predict and to correct for these effects.

**Fig. 13:**
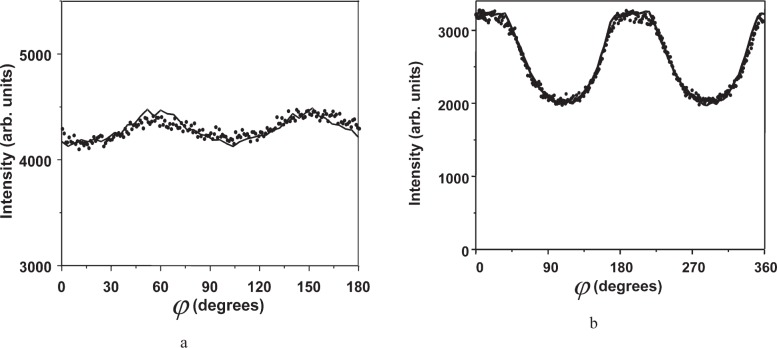
Accounting for the actual specimen shape: observed (dots) and modelled (line) intensity variation by rotating (a) a 14 mm × 14 mm square specimen and (b) a 22 mm × 8 mm rectangular specimen. The specimen was made of tungsten, and the observation refers to the (110) reflection, *ψ* = 60°, 2*θ* = 40.26°. Beam size 4 mm × 4 mm. Measurement conducted on MRD I.

Figure 13 shows the *ϕ* scan (at *ψ* = 60°) for a square (14 mm × 14mm, (a)) and a rectangular (22 mm × 4 mm, (b)) tungsten specimen. Both diagrams follow a similar pattern with a 180° repetition period; this is a clear effect of the particular shape of the specimen. For a specimen bigger than the (footprint of the) incident beam or for a rotationally symmetric specimen, the intensity should be a constant, the specimen always being illuminated during the measurement. Should the specimen possess an odd shape and should the beam footprint on the specimen surface be smaller than the specimen surface itself, then during a *φ* scan a varying portion of the surface will be bathed by the beam. For a rectangular specimen, in particular, the illuminated area is expected to be maximum when beam and specimen diagonal are aligned and minimum when the smaller side of the specimen is aligned with the beam. Measurement and raytraced data agree quite well and confirm the expected result. The small differences can be ascribed to edge effects not considered in detail here. In particular, the specimens were not perfectly rectangular (edges are rounded) and the effect of the penetration on the near-edge regions hasn’t been considered.

### 5.2 Fitting and Interpolation of the Raytraced Data

The main drawback of the Monte Carlo procedure is the slow calculation speed. For a given system, a possible way to get a quicker evaluation of the correction curve is to model the Monte Carlo data using a technique analogous to the “experimental” correction proposed by Welzel and Leoni [19]. The gain is both in speed (there is no need for experimental measurements) and flexibility (it can be easily adapted to new conditions). Moreover, better accuracy is obtained with respect to any other correction method based on simplified or empirical formulae. Since the variation in the peak parameters with respect to the angular parameters of the system are quite smooth, a linear or a cubic spline interpolation can be successfully used to access regions for which the correction has not been calculated.

### 5.3 Aberrations in Stress Analysis

Extensive literature exists on the determination of the residual stress state in the surface and sub-surface regions of the most diverse materials (see, e.g., Refs. [17,18]). The aberrations influencing the accuracy of stress data are those modifying the position of the diffraction peaks (quite common in the Bragg-Brentano geometry, due to specimen positioning, tilting, flat surface etc.). Intensity aberrations can be neglected, playing a major role in texture determination, whose effect on the stress evaluation is, in most cases, of second order. Intensity, on the other hand, can be a serious problem at high tilting when only a small fraction of the signal reaches the detector, seriously affecting the signal/noise ratio.

In most literature work, however, instrumental effects (in particular peak shift) are not taken into account or not (explicitly) corrected for. A comparison between pinhole and polycapillary collimators in parallel beam geometry (cf. Ref. [40]), has shown corrections to be necessary only when the former are used. The proposed raytracing algorithm can be used to obtain correction curves for instrumental effects in *θ*/2*θ* scans at different tilt *ψ* traditionally used for stress analysis and to validate the findings of Scardi et al. [40].

**Fig. 14:**
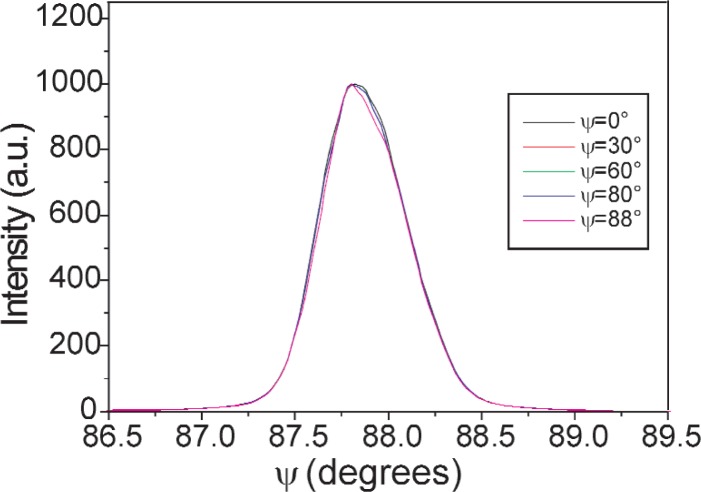
Simulation of the (321) reflection of the NIST SRM660a LaB_6_ standard on MRD II: peak position does not vary tilting the specimen (for clarity the curves have been normalised). Analogous results can be obtained for negative tilting. Beam size 2 × 2 mm, specimen size 20 × 20 mm.

**Fig. 15:**
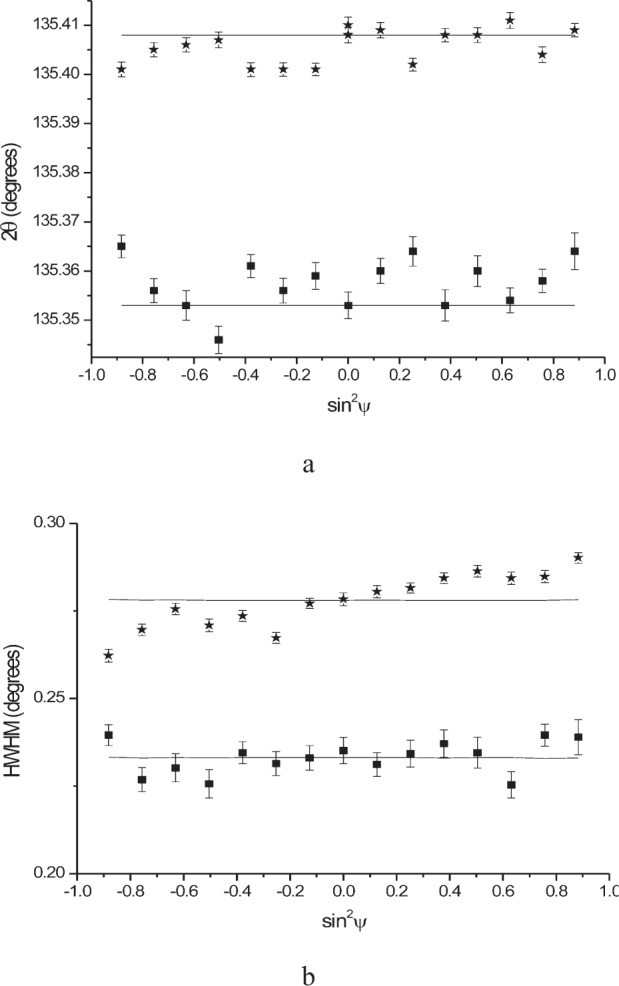
Trend of peak position (a) and Half Width at Half peak Maximum (HWHM) (b) versus specimen tilting for the 422 reflection of gold. Data collected on MRD II (squares = 1 × 1 mm slits opening, stars = 4 × 4 mm slits opening) are taken from Scardi et al. 2000 (cf. Fig. 4 in cited paper). Simulations are shown as continuous lines (for clarity, data shifted as in the cited paper).

Modeling shows absence of instrumental effects within the accessible angular range. Shape, width and position of diffraction peaks are not influenced by the tilting (Fig. 14) i.e., instrumental effect on the sin^2^*ψ* plot are absent or negligible. As also clear from Fig. 15, simulations are in good agreement with the data of Ref. [40]. Non-perfect parallelism in the beam and specimen displacement/shape effects, however, could cause a fictitious shift in the peak position.

### 5.4 Instrumental Function and Influence of the Optical Devices on the Profile Shape

Besides introducing possible variations in intensity, peak shape and peak position, optical components affect the width of the reflection as commonly seen in a *θ*/2*θ* scan. Possible factors influencing this phenomenon have already been extensively studied for traditional Bragg Brentano diffractometers [26]. For a parallel beam setup such as the one considered here, the expected variation is limited because of the intrinsically high divergence of the beam (the problem is more critical on high resolution diffractometers).

Two specimens have been used to characterize the instrumental function, namely a NIST standard (LaB_6_, NIST SRM 660a, Ref. [21]) and a commercial ZnO powder. The SRM 660a is certified for line position and absence of specimen-related broadening, but ZnO is a valid alternative when the resolution of the instrument is not particularly high. Moreover, unlike lanthanum hexaboride, ZnO permits the preparation of specimens showing very flat surfaces and high resistance to handling (e.g., they can be employed for measurements at negative *ψ* tilting (powder upside-down)).

**Fig. 16:**
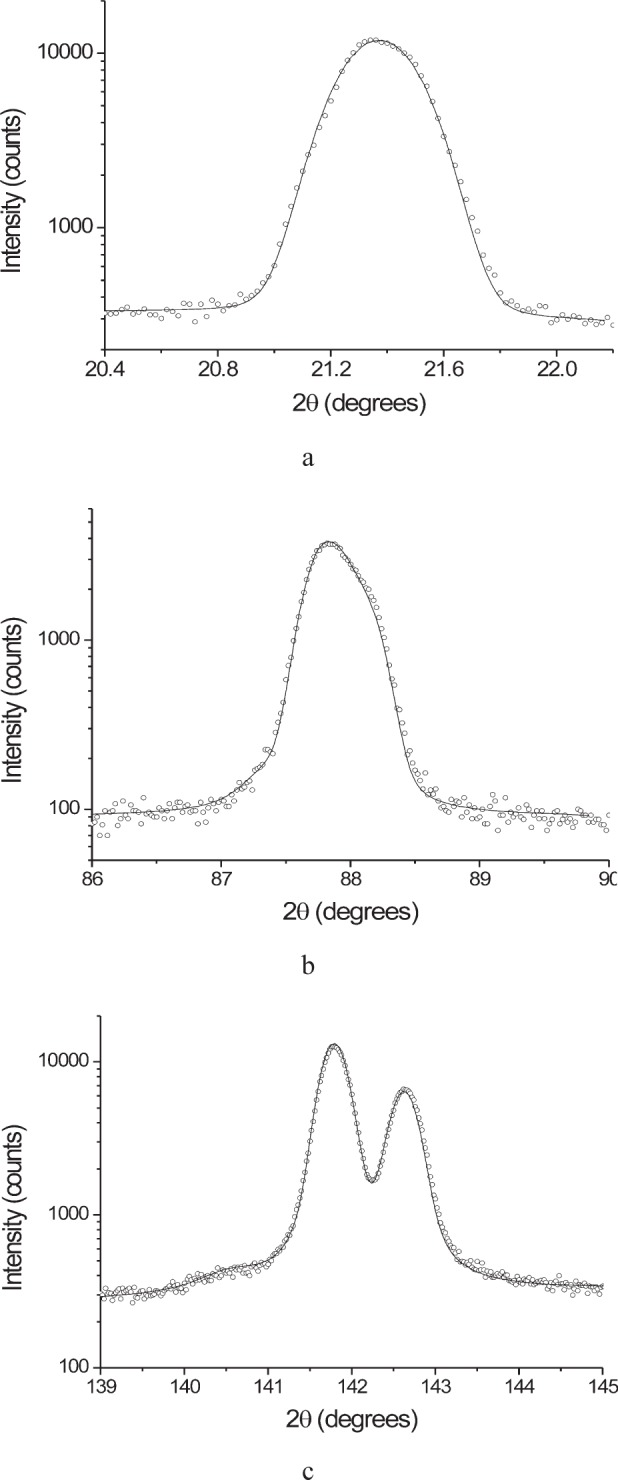
Instrumental profiles for MRD II measured using the SRM660a LaB_6_ standard. The 100, 321 and 510 reflections are shown in (a),(b),(c), respectively. The specimen was square (2 × 2 cm) and was mounted on an aluminium frame; beam size was limited to 2 × 2 mm. Open dots correspond to raw data, whereas continuous line is the result of simulation.

Figure 16 shows a set of LaB_6_ reflections collected on MRD II and the corresponding modeling results. The raytraced profile well approximates the measured peak both at low and high 2*θ* angle. We should bear in mind that the simulation was conducted using nominal values for the instrument dimensions, thus the model parameters are not optimized. To fill the minimal gap between experiment and simulation, fitting of the model equations on the experimental data would be necessary.

**Fig. 17:**
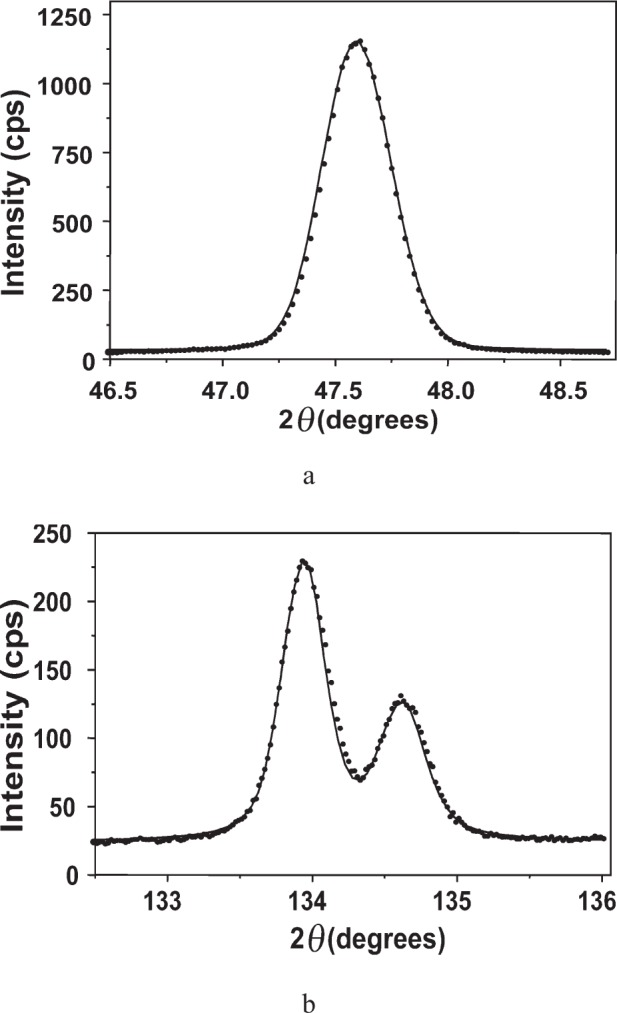
Comparison between measurement on the MRD I instrument (dots) and simulation (line). (a) (102) reflection and (b) (205) reflection of a zinc oxide powder. Measurement and simulation conditions were: incoming beam 4 × 4 mm and specimen size 14 × 14 mm.

Figure 17 shows an analogous measurement conducted on ZnO. As expected for the relatively low resolution of these optics, the differences between zinc oxide and SRM 660a (Fig. 16) are negligible. Considering that a complete distribution of wavelengths was not used in the primary beam, (we use a single wavelength in the raytracing and we convolve the raytraced profile with the emission profile) the agreement between model and simulation is rather good; the low angle reflections are a bit broader than expected, whereas the opposite is true for the high angle ones (cf. Figs. 17a and 17b). Accounting for the correct wavelength dispersion, would probably correct for this discrepancy.

## 6. Conclusions

A procedure has been presented for the raytracing of a parallel-beam diffractometer. The emphasis has been placed on the analysis of an instrument possessing a polycapillary collimator on the primary path. The proposed algorithm permits the evaluation of the instrument response in various diffraction modes. In particular, instrumental effects such as the variation of intensity and peak position, as well as the dependence of the profile shape on the diffraction angle can be easily obtained. The algorithm is quite flexible and can be easily adapted to any diffractometer.

Corrections for pole figure measurement, residual stress analysis, and traditional *θ*/2*θ* diffraction experiments are proposed and tested against measured data. Excellent agreement is found even in regions where traditional simplified models fail.

Raytracing and instrumental modeling represents a valid tool for experiment planning, providing a prediction of the instrumental response and accounting for possible aberrations and artifacts.

The results of the present work will serve as a basis for the analysis of 2-dimensional diffraction maps (e.g., reciprocal space maps and stress/texture maps).

## Figures and Tables

**Table 1:** Modeling result: *R* is the geometrical radius of the lens, *w_I_* the limit for *region I* in the ideal case, *R*model and *σ*model the effective radius of the lens and the HWHM of the Gaussian distribution function [Eq. (3)], respectively. The agreement coefficient R_fit_^2^ are also reported

Diffractometer	*R* (mm)	*w_I_* (mm)	*R*_model_ (mm)	*σ*_model_ (mm)	*R*_fit_^2^
MRD 1	3.5	4.95	3.11(2)	1.78(1)	0.99993
MRD 2	4.5	6.36	4.20(2)	2.24(1)	0.99998

**Table 2:** Results for the modeling of the data shown in Fig. 8

	Expected dimension	Refined dimension	*R*_fit_^2^
Fixed height	0.1	0.0947(2)	0.99934
Fixed width	0.1	0.0978(3)	0.99945

**Table 3:** Parameters characterizing the diffraction system

Parameter	Description
SW	Width of the specimen
SH	Height of the specimen
CW	Width of the entrance section of the parallel foils collimator
CH	Height of the entrance section of the parallel foils collimator
CD	Distance between the parallel foils
CT	Thickness of one of the foils composing the collimator
AW	Width of the analyzer crystal
AH	Height of the analyzer crystal
*d*_11_	Distance between the exit of the lens and the goniometric center
*d*_21_	Distance between the goniometric center and the entrance section of the parallel foils collimator
*d*_22_	Length of the parallel foils collimator
*d*_23_	Distance between the exit of the parallel foils collimator and the center of the crystal analyzer
*d*_24_	Distance between the center of the crystal analyzer and the detector
*RD*	Radius of the sensitive area of the detector
*θ*	Primary angle (angle in the equatorial plane between the surface of the specimen and the axis of the lens)
*θ*_B_	Bragg (diffraction) angle relative to the Kα_1_ wavelength for the reflection considered
*θ*_d_	Secondary angle (angle in the equatorial plane between the secondary arm and the surface of the specimen, considered from the negative y direction of **G**)
*θ*_a_	Angle of the analyzer. In our case, since the {002} reflection of graphite is used, it corresponds to 13.285°
*σ*_a_	Mosaic spread of the analyzer, i.e., HWHM of the rocking curve collected on the employed reflection of the crystal

## Footnotes

1 Certain commercial equipment, instruments, or materials are identified in this paper to foster understanding. Such identification does not imply recommendation or endorsement by the National Institute of Standards and Technology, nor does it imply that the materials or equipment identified are necessarily the best available for the pupose.

2 The result can be considered as qualitatively valid only. Non-linear effects, mainly due to the processing of the x-ray film, hinders a reliable quantitative analysis.

3 A Cartesian representation of the RIDF has been obtained by introducing the radial coordinate $\rho \left(x,y\right)=\sqrt{x^{2}+y^{2}}$. The error function is defined as $\text{Erf}\left(x\right)=\frac{2}{\sqrt{\pi }}\int_{0}^{x}e^{-t^{2}}dt$.

4 We define *goniometric center* the intersection between the *equatorial plane* (the horizontal plane passing through the middle of source and detector) and the *goniometric axis* (the axis of rotation of the goniometer).
